# “Adverse event reports of seizure for insomnia medication from 1967 to 2023”. The challenge of using hypnotic medication in people with epilepsy

**DOI:** 10.3389/fneur.2026.1727350

**Published:** 2026-03-03

**Authors:** Giovanni Di Mauro, Claudio Liguori

**Affiliations:** 1 Neurology Unit, University Hospital of Rome Tor Vergata, Rome, Italy; 2 Department of Systems Medicine, University of Rome Tor Vergata, Rome, Italy

**Keywords:** dual orexin receptor antagonist, epilepsy, hypnotics and sedatives, insomnia, melatonin

We've read with remarkable interest the paper “*Adverse event reports of seizure for insomnia medication from 1967 to 2023*” published on *Scientific Reports* in July 2025, in which Authors investigated the risk to induce seizures of different drugs used for the treatment of insomnia (1).

In this study, the Authors reported the results of the American Insomnia Survey done in 2020 (in which up to 22% of responders met the criteria for insomnia) and highlighted how clinicians very frequently tend to treat insomnia with hypnotic medications, especially in consideration of the lack of accessibility to cognitive behavioral therapy for insomnia (CBT-i) (2). The common practice to prescribe hypnotics happens despite the lack of robust randomized trials, that must always be conducted to minimize drug-related side effects. The authors reviewed a vast dataset deriving from the reports of adverse events related to medications used for the treatment of insomnia from 140 countries between 1967 and 2013, evaluating the seizure risk associated with these medications measured as reported odds ratio (ROR). They found that seizures were significantly associated with various hypnotic medications including benzodiazepines, Z-drugs, antidepressants, melatonin, atypical antipsychotics and first-generation H1 antagonists. No association was found between seizures risk and melatonin receptor agonists and dual orexin receptor antagonists (DORAs).

The presented study is one of the most valuable in the field, in considerations of the huge number of case reports and adverse events reviewed. Nevertheless, the authors do not explore the underlying pathophysiological mechanisms useful to explain why some drugs are associated to seizure risk (like benzodiazepines, Z-drugs and antipsychotic) and others are not (like DORAs). Moreover, the Authors did not discuss about the controversial results regarding the increased risk for seizures of melatonin and the lack of association between melatonergic receptor agonists and seizure occurrence.

Insomnia is a high common sleep disorder in the general population. Chronic insomnia may affect approximately 10%−12% of adults, with an additional 15%−20% reporting occasional insomnia symptoms (3–7, 33). When broader definitions are used, such as any insomnia symptoms or sleep dissatisfaction, prevalence rates can reach up to one-third of adults (8, 33).

Translating the results of the study in the common clinical practice, the importance of these findings also regards the frequency of sleep disorders in people with epilepsy (PwE) and the importance of promoting sleep without increasing the risk of seizures in PwE.

Therefore, to complete the discussion of the study “*Adverse event reports of seizure for insomnia medication from 1967 to 2023*,” we do believe that both preclinical and clinical evidence should be cited in order to explain the Authors' findings.

Orexin (hypocretin) neurons, located in the lateral hypothalamus, play an extensive role in the regulation of the sleep–wake cycle. During wakefulness it can be registered the highest orexinergic activity, which then decreases during slow-wave sleep and is virtually absent during rapid eye movement (REM) sleep (9). This circadian rhythm mirrors oscillations in the activity of cortical ad subcortical brain structures. The high firing of orexinergic neurons stabilizes wakefulness through cortical desynchronization, on the contrary a weak orexinergic activity consolidates slow-wave sleep enhancing the thalamocortical synchronization (10). Since seizures are often associated with transitions from wakefulness to sleep and vice versa, sleep stability should be targeted for preventing the risk for nocturnal seizures. Moreover, sleep exerts beneficial effects on epileptogenesis and therefore maintaining the sleep-wake cycle regularity and the regulation of the physiological sleep are both targets for reducing the risk for seizures in PwE. According to this, we may hypothesize that the pharmacological antagonism to orexin receptors, exerted by DORAs, might reduce the risk of seizures. Accordingly, both preclinical and clinical studies suggests that DORAs do not increase seizure risk and may play an anticonvulsant role instead.

Experiments in animal models showed, indeed, that orexinergic agonism produces proconvulsant effects and conversely orexinergic antagonism, that mirrors the neuronal silence observed in REM sleep (a state characterized by low seizure susceptibility) exerts anticonvulsant effects. The direct administration of orexin-A and orexin-B into the cortex increases epileptiform activity, measured in terms of spike number, amplitude, and EEG spectral power, as well as seizure-related motor manifestations. (11) At the cellular level, orexins enhance hippocampal neuronal excitability and increase excitatory neurotransmitter release (12). In experimental rats exposed to proconvulsant agents such as pilocarpine, pentylenetetrazol (PTZ), and maximal electroshock induced seizures, it has been demonstrated an upregulation in orexin expression in the hippocampus (13, 14). In contrast, both orexin-1 receptor (OX1R) antagonists (e.g., SB-334867) and orexin-2 receptor (OX2R) antagonists (e.g., TCS OX2 29) attenuate seizure severity and duration in PTZ models when administered intrahippocampally or intracerebroventricularly (15).

DORAs, including suvorexant, an FDA-approved therapy for insomnia, significantly reduce seizure duration in PTZ mice. Suvorexant may exert additional modulatory effects via GABA-A and glutamate receptors (16). Furthermore, in sleep-deprived Wistar rats, both OX1R and OX2R antagonists delayed seizure onset, shortened seizure duration, and reduced mortality following PTZ exposure, indicating that orexin antagonism may protect against the exacerbation of seizures induced by sleep deprivation (17).

There is no substantial literature helping the translation of orexin biology into epilepsy clinical practice. Only some observational studies documented the clinical potential of improving sleep and treating insomnia disorder in PwE by using DORAs (18).

Collectively, animal data strongly suggest that orexinergic signaling facilitates neuronal hyperexcitability and seizure generation, whereas orexin receptor antagonism mitigates these processes. Clinical studies are needed for understanding the clinical potential of DORAs in improving sleep and treating the comorbid insomnia disorder in PwE.

Regarding the role of melatonin and melatonergic receptor agonism, globally the literature describes their safety in PwE with no evidence of seizure exacerbation and, in some cases, seizure reduction in patients with comorbid insomnia disorder and refractory epilepsy (19–23). Melatonin is a hormone secreted by the pineal gland reflecting the circadian activation of the suprachiasmatic nucleus, which acts as a biological clock and receives the photic input directly from the retina through the retino-hypotalamic tract (24). Melatonin exerts its effects binding the high-affinity G-protein-coupled receptors MT1 and MT2 broadly expressed in the brain and regulates sleep-wake cycle, circadian rhythm as well as epileptogenesis (25). Circadian fluctuations of melatonin levels influence neuronal activity of different brain networks. An increase in melatoninergic tone enhances neurons synchronization, reduces glutamatergic neurotransmission and increase GABA-ergic signaling. A reduced melatonin activity can increase hyperexcitability and neuronal firing rate instead (26). Mechanistically, melatonin's antioxidant, anti-inflammatory, and neuroprotective properties are proposed to contribute to its potential anticonvulsant effects (27, 28).

Melatonin anticonvulsant properties have been highlighted in preclinical studies conducted in animals. In a study from Savina and colleagues, melatonin produced a sustained anticonvulsant effect, increasing latency and decreasing the severity of audiogenic seizures in rats (29). Moreover the acute iv administration of high dose of melatonin significantly elevated the clonic threshold of convulsions induced by pentetrazole and completely abolished kainated-induced seizures in mice (30, 31). Hence, it can be hypothesized that melatonin itself and melatoninergic agents do not have an unfavorable profile of action in terms of increasing seizures risk or worsening epilepsy control. The primary hypothesis linking melatonin, but not melatonin receptor agonists, to an increased seizure risk relates to the sleep synchronization induced by this neurohormone. Accordingly, sleep synchronization—and the subsequent increase in Non-REM sleep—may predispose susceptible individuals to seizures. However, this remains a hypothesis based on a preclinical rationale and is not yet supported by evidence in clinical practice.

The translation of both these mechanistic considerations into the therapeutic decision-making process must reflect the evidence from regulatory approval and international guidelines (32). DORAs, thanks to their highly targeted action, without enhancing GABAergic neurotransmission, are globally indicated as a safe treatment of chronic insomnia as documented in randomized clinical trials. Therefore, they are the drug of choice in PwE as well. Melatonin receptor agonists are not uniformly approved worldwide for the treatment of insomnia and only melatonin 2 mg prolonged release is approved for treating chronic insomnia in several regions in a restricted range of people (aged >55 y.o.), although it is widely used as supplement for insomnia complaints and circadian sleep-wake rhythm disorders. Hence, randomized clinical trials are needed for considering the favorable profile of melatonergic drugs in PwE.

In conclusion, DORAs used in the treatment of chronic insomnia do not increase seizure risk and preclinical findings support an anticonvulsant potential of orexin antagonism, possibly through facilitation of inhibitory over excitatory neurotransmission. Literature reports controversial data regarding the use of melatonin and melatonergic agonists and therefore further evidence is needed.
